# Genome-Wide Loss of Heterozygosity and DNA Copy Number Aberration in HPV-Negative Oral Squamous Cell Carcinoma and Their Associations with Disease-Specific Survival

**DOI:** 10.1371/journal.pone.0135074

**Published:** 2015-08-06

**Authors:** Chu Chen, Yuzheng Zhang, Melissa M. Loomis, Melissa P. Upton, Pawadee Lohavanichbutr, John R. Houck, David R. Doody, Eduardo Mendez, Neal Futran, Stephen M. Schwartz, Pei Wang

**Affiliations:** 1 Program in Epidemiology, Division of Public Health Sciences, Fred Hutchinson Cancer Research Center, Seattle, Washington, United States of America; 2 Department of Otolaryngology–Head and Neck Surgery, University of Washington, Seattle, Washington, United States of America; 3 Department of Epidemiology, University of Washington, Seattle, Washington, United States of America; 4 Program in Biostatistics and Biomathematics, Division of Public Health Sciences, Fred Hutchinson Cancer Research Center, Seattle, Washington, United States of America; 5 Department of Pathology, University of Washington, Seattle, Washington, United States of America; 6 Clinical Research Division, Fred Hutchinson Cancer Research Center, Seattle, Washington, United States of America; 7 Department of Genetics and Genomics Sciences, Mt. Sinai School of Medicine, New York, New York, United States of America; Baylor College of Medicine, UNITED STATES

## Abstract

Oral squamous cell cancer of the oral cavity and oropharynx (OSCC) is associated with high case-fatality. For reasons that are largely unknown, patients with the same clinical and pathologic staging have heterogeneous response to treatment and different probability of recurrence and survival, with patients with Human Papillomavirus (HPV)-positive oropharyngeal tumors having the most favorable survival. To gain insight into the complexity of OSCC and to identify potential chromosomal changes that may be associated with OSCC mortality, we used Affymtrix 6.0 SNP arrays to examine paired DNA from peripheral blood and tumor cell populations isolated by laser capture microdissection to assess genome-wide loss of heterozygosity (LOH) and DNA copy number aberration (CNA) and their associations with risk factors, tumor characteristics, and oral cancer-specific mortality among 75 patients with HPV-negative OSCC. We found a highly heterogeneous and complex genomic landscape of HPV-negative tumors, and identified regions in 4q, 8p, 9p and 11q that seem to play an important role in oral cancer biology and survival from this disease. If confirmed, these findings could assist in designing personalized treatment or in the creation of models to predict survival in patients with HPV-negative OSCC.

## Introduction

Oral squamous cell cancer (OSCC), which includes malignancies of the oral cavity and the oropharynx, proves fatal in many instances. The tumor and/or its treatment often lead to orofacial dysfunction and disfigurement. Oral cavity cancers are largely caused by tobacco and alcohol use, while infection with oncogenic Human Papillomavirus (HPV) often plays a role in the genesis of oropharyngeal cancer. Patients with HPV-positive oropharyngeal tumors generally have better treatment response and survival than do patients with HPV-negative oropharyngeal tumors. HPV status in oral cavity tumors does not seem to impact treatment response and survival. It is unclear whether this is a reflection of the underlying biology of oral cavity tumors, or rather because the low frequency of HPV-positive tumors in oral cavity cancer patients (reported to be 5–15%) has prevented a robust investigation. HPV-negative OSCC patients with tumors of the same clinical and pathologic stage have a heterogeneous response to treatment and likelihood of recurrence and survival. However, the molecular basis for this heterogeneity is also largely unknown.

Loss of heterozygosity (LOH) and DNA copy number aberration (CNA, defined as having an altered DNA copy number at a specific locus in the tumor compared to that in the peripheral blood leukocytes) that are associated with the inactivation of tumor suppressor genes (TSG) and the activation of oncogenes occur with various frequencies in squamous cell carcinoma of the oral cavity and oropharynx (OSCC) and other types of squamous head and neck cancer (HNSCC) [1, 2]. LOH and CNA patterns appear to differ at different points in the natural history of OSCC, and these patterns are, to some extent, correlated with clinical disease outcomes [3]. Furthermore, there is some evidence to suggest that LOH/CNA may be superior predictors of disease outcomes compared to the traditional TNM staging system [4, 5]. There is evidence that LOH on 2q, 3p, 6q25-27, 8p, 8p21.2, 8p23, 9p21-22, 10q, 11q23, 13q, 14q, 17p or 18q are associated with recurrence and/or poor survival of OSCC and/or HNSCC patients [6–9]. Other studies showed that DNA amplification, and DNA copy number gains and losses predict recurrence and/or survival of OSCC and/or HNSCC patients [6, 10–24]. Array CGH (aCGH) studies have reported that HPV-positive and HPV-negative HNSCC have both common and distinct CNA [25], and have also observed that gains and losses at various chromosome arms are associated with recurrence and/or length of survival [4, 5, 26, 27].

Although these efforts greatly advanced our understanding of genetic alterations relating to oral cancer, signal contamination caused by the presence of non-malignant cell populations, the low resolution, and the inability to identify balanced chromosomal changes such as copy-neutral LOH [28, 29] where LOH in one allele is compensated by the copy gain in the alternate allele in the paired chromosome, are the major limitations to the above mentioned studies. To improve upon these aspects in our efforts to identify potential chromosomal changes that may be associated with survival in HPV-negative OSCC, which accounts for the majority of OSCC worldwide, we interrogated paired peripheral blood DNA and DNA from tumor cells isolated by laser capture microdissection (LCM) using Affymetrix Human SNP Array 6.0 to examine the genome-wide landscape of LOH and CNA and to explore whether LOH and CNA are associated with OSCC-specific mortality.

## Materials and Methods

### Ethics statement

This work was conducted with written informed consent of study participants and was approved by the Institutional Review Offices of the Fred Hutchinson Cancer Research Center and the Veterans Puget Sound Healthcare System.

### Study population

Eligible study participants were those who were ≥18 years of age, could communicate in English, were without prior treatment with radiation and/or chemotherapy, who underwent surgical resection or biopsy at the University of Washington Medical Center, Harborview Medical Center or the VA Puget Sound Health Care System in Seattle, Washington for their first primary OSCC between 2003 and 2010 [30]. Eligible participants were asked to donate tumor tissue and peripheral blood at or before the time of resection or biopsy. Tumor DNA was tested for HPV as previously described [31]. Participants were interviewed in person regarding demographic, lifestyle (including tobacco and alcohol use), medical, functional, and quality of life information. Tumor characteristics were obtained from medical records. Patients were followed actively through periodic telephone interviews to ascertain recurrence and changes in lifestyle characteristics, and passively through medical record reviews and linkages to the U.S. Social Security Death Index and the FHCRC’s Cancer Surveillance System (one of NCI’s SEER registries), which is updated semi-annually with the Washington State Death Certificate database and annually with the National Death Index. Classification of death as due to OSCC was based on information on the medical records and death certificates and independent adjudication by two otolaryngologists. This study was conducted with written informed consent and approvals by the Institutional Review Boards of the Fred Hutchinson Cancer Research Center, University of Washington and the VA Puget Sound Health Care System.

### Isolation of tumor cells by laser capture microdissection (LCM)

Tumors retrieved from liquid nitrogen storage were embedded on dry ice using Tissue-Tek OCT Compound (Sakura Fineteck U.S.A., Torrance, CA) in 2-methylbutane. A 10-μm section was stained with hematoxilin and eosin for the pathologist (MP Upton) to identify regions rich in tumor. The same tumor-rich regions in successive sections were isolated by LCM using an Arturus^XT^ Microdissection System (MDS Analytical Technologies, Sunnyvale, CA) to yield enough tumor cells for 500 ng DNA for LOH/CNA detection.

### Isolation of DNA from paired tumor cells and peripheral white blood cells

DNA from tumor cells was extracted using the Qiagen DNA Micro Kit; DNA from the white blood cells was isolated by salt precipitation [32]. The purity of DNA was high as judged by spectrophotometric A260 to A280 ratio of ≥1.8.

### Interrogation of LOH and CNA using Affymetrix Genome-wide Human SNP Array 6.0

DNA samples were further processed per Affymetrix protocols and interrogated using the Affymetrix 6.0 SNP array. Affymetrix software tool and Genotyping Console 3.0.2 were used to determine signal intensities and whether samples passed the Affymetrix QC threshold of Contrast QC> 0.4 and a QC Call Rate>86%. The respective corresponding values for our 75 samples were 1.87 and >97.1%. Genotype calls were made using Birdseed v2 (Affymetrix Power Tools (APT), http://media.affymetrix.com/). Across the 19 replicate arrays of a reference sample assayed in different batches, ~98.8% of the probes had identical SNP calls. For quality control, we filtered out 3% SNPs that, in majority of subject DNA samples had poor APT confidence scores, or had high genotyping call discrepancy over the 19 reference arrays. To normalize raw allele intensity, we used the R package AROMA [33], which can adjust for allelic crosstalk, probe affinity, PCR fragment length and probe sequence effects. The Affymetrix.cel files and associated patient characteristics can be found in the GEO database with Accession No.GSE68717.

### Statistical methods for inferring LOH and CNA based on genotype calls and normalized probe intensities

We inferred the LOH status at each locus of each patient based on the genotype calls at that locus from the paired tumor and blood samples. We excluded any SNP that was homozygous in all tumors, which are non-informative to infer LOH, leaving 223,357 SNPs for further analyses. LOH positive loci may have copy number loss (CN ≤ 1), be copy number neutral (CN = 2) or show copy number gain (CN ≥ 3). LOH negative loci may also show copy number loss, gain, or neutrality. To determine the CNA segment, we used Parent/allele specific copy number estimation method (R package pscn [34]) which smoothes and estimates DNA copy number using maximizing likelihood and segments the results into regions with constant copy number. The 900K CNA probe data were thus downsized to 131K segments. Then based on the segmented data, we use the paired tumor and blood samples to estimate the FDR on CNV gain/loss calls in tumor samples at a given magnitude cutoff. Specifically, we assumed that there is no true copy number variation in the blood sample, and estimated FDR as the ratio of the number of probes surviving the cutoff in the blood sample versus that in the tumor sample. The final CNA gain/loss calls were made by controlling the FDR of the results at an average targeted level of 10% (the FDR across subjects ranged from 0 to 12%, with a median of 0.96%).

To assess the association between individual SNP’s LOH/CNA status and patient survival, we focused on OSCC-specific death (n = 24) and employed a multivariable Cox regression model adjusting for age, sex, and smoking history to evaluate the strength of the associations. The association of LOH/CNA events with OSCC-specific survival was determined with hazard ratios (HRs), and the corresponding 95% confidence intervals (CIs). There are possible models including univariate model with CNA only, univariate model with LOH only, multivariate model with both CNA and LOH, and the full model with CNA, LOH and a term representing the multiplicative interaction of CNV and LOH (CNA*LOH). Sex, age and smoking status were included in all the above models. We conducted the log-likelihood ratio test comparing the full model and the nested model to find out the significance of association. To account for multiple hypotheses testing, we further estimated False Discovery Rate using the R package q-value [35].

## Results

We generated genome-wide LOH and CNA data on 75 patients with first, newly-diagnosed HPV-negative OSCC. S1 Table shows selected characteristics of these patients who were mostly white, male, current smokers and alcohol drinkers. The median follow-up was 75.7 months in patients alive at last follow-up (range 32.7–111.4), and 46.9 months (range 1.1–111.4) for all 75 patients. About 58.7% had late stage (AJCC stage III & IV) OSCC and 46.7% had lymph node involvement. Forty patients were deceased as of March 2014, among whom 24 patients had died of OSCC.

### Genome-wide LOH events and copy number gains/losses in tumor tissue

**Fig 1:**
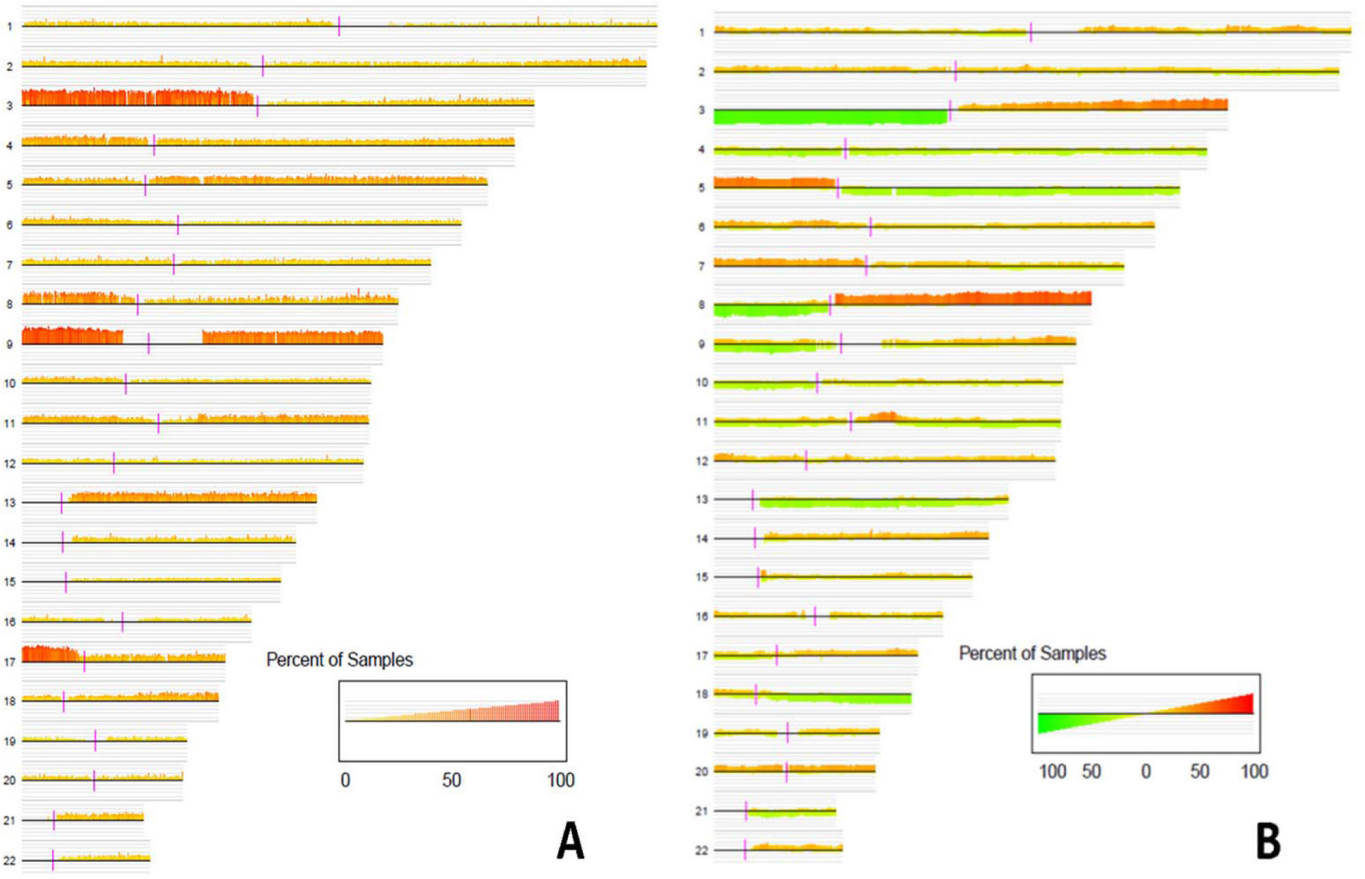
Consensus plot of genome-wide LOH events. (A) and copy number gains and losses (B) for each autosome. The height and the color of the vertical lines represent the percentage of samples in which the corresponding probes have LOH (A) or copy number gains (red)/losses (green) (B). Horizontal gray lines indicate percentages ranging from 0, 20%, 40%, 60%, 80% to 100% The p arm (left) and q arm (right) in each chromosome are delineated by a vertical purple bar.

The percentage of samples showing LOH at each probe from autosomes ranged 0%-90% (mean ± sd: 14.2 ± 13.3%) (Fig 1A). Frequent LOH events (appearing in >20% of the samples) were observed in all autosomes. Some segments on 3p and 9p along with one SNP in 8q24 (rs16904097) and one SNP in 17p (rs2042004) exhibit extremely frequent LOH (in >80% of samples) (Fig 1A). The region on Chr. 9p with extremely frequent LOH contains 30 SNPs, corresponding to eight genes (*FLJ35024*, *KDM4C*, *CCDC171*, *IL33*, *PTPRD*, *TTC39B*, *SH3GL2*, *FREM1*) and 15 inter-genic positions (rs1947447, rs10976390, rs1538718, rs10757623, rs7864275, rs4512431, rs1328001, rs10967005, rs10733410, rs4740766, rs12346508, rs726353, rs10125418, rs2375075, rs882092, rs10811970) within 9p21.2-9p24.3. The neighboring region of these 30 SNPs harbor several known TSG, including *p16*/*CDKN2* gene cluster, *TUSC1*, *SH3GL2* and *DMRT2*, which also exhibit frequent LOH events: one SNP in *p16* showed LOH in 37% of the samples; 20 SNPs in *SH3GL2* showed LOH in 36% of the samples, and one SNP in *DMRT2* showed LOH in 41% of the samples. The respective top 100 SNPs showing the most frequent LOH (including copy number gain LOH copy number loss LOH and copy number neutral LOH), DNA copy number gain, and DNA copy number loss are listed in S2 Table.

Frequent copy number gain events (appearing in >20% of the samples) were observed in 20 autosomes (except Chr. 4 and 21, Fig 1B); while frequent copy number loss events (similarly defined) were observed in 14 autosomes (except Chr. 1, 6, 7, 12, 16, 19, 20, 22). Among oncogenes and TSG that have been implicated in HNSCC, we found copy number gain and loss of *EGFR* (36% and 1.3% of the samples, respectively), *ERBB2* (21.3% and 4% of the samples, respectively), *FAT1* (5.3% and 27% of the samples, respectively), *SMAD4* (1.3% and 41% of the samples, respectively); and *CDKN2A* (8% and 54.7% of the samples, respectively).

Percentage of probes in 22 autosomes showing CNA and LOH events varies among different samples (S1 Fig). The percentage of probes showing LOH in each sample ranges 0.33% to 43% (mean ± sd: 14.19 ± 10.75%). The percentage of probes showing CNA in each sample ranges from 0.03% to 82.6% (mean ± sd: 30.1% ± 18.4%). Copy number gain events range from 0% to 52% (mean ± sd: 17.1% ± 11.6%); copy number loss ranges from 0% to 33.9% (mean ± sd: 13 ± 9.1%). Tumors with more LOH also are more likely to have more CNA.

**Fig 2:**
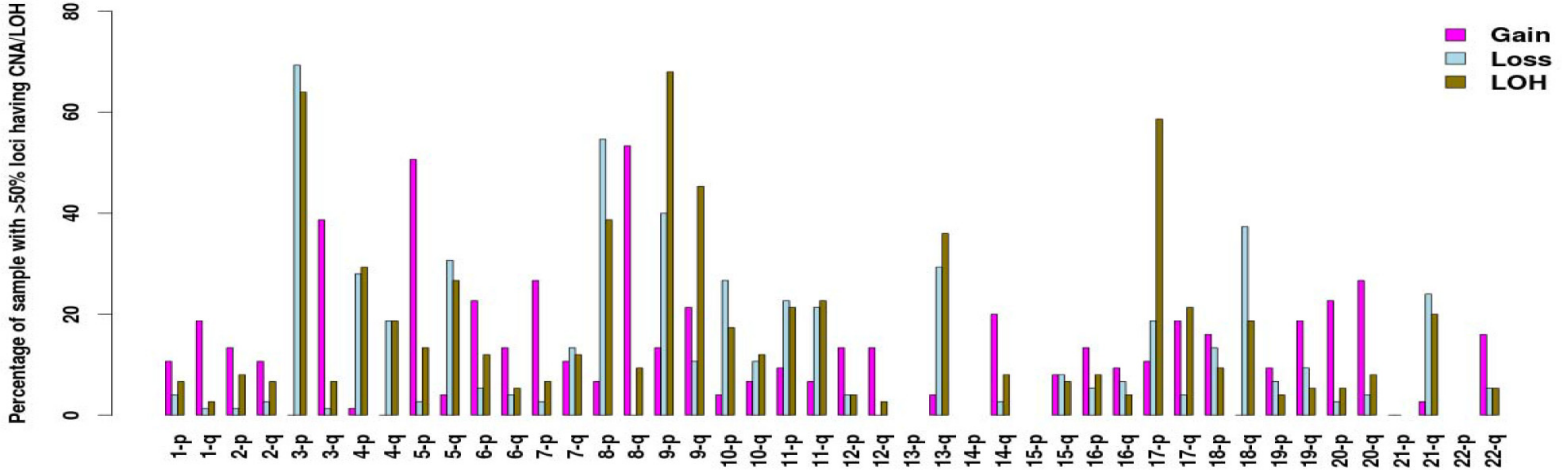
Percentage of OSCC having large-region-gain/loss/LOH for each chromosome arm. Large-region-gain/loss/LOH is defined as gain/loss/LOH events occurring at more than 50% of probes of one chromosome arm in a sample.

Chromosome arms 3p, 8p, 9p, 9q, 13q and 17p contain *large-region-LOH* events, as defined by having more than 50% of probes on the chromosome arm showing LOH, in at least 27 of the 75 samples (36%) (Fig 2). Similarly, frequent *large-region-gain* was seen for 3q (38.7% of the samples), 5p (50.7%), and 8q (53.3%), and frequent large-region-loss was seen in 3p (69.3%), 8p (54.7%) and 9p (40%).

### Association of genome-wide LOH/CNA events with tumor characteristics, lifestyle factors and OSCC-specific mortality

We clustered samples into two groups using genome-wide autosome CNA and LOH events and examined the association between the resulting clusters and tumor characteristics, OSCC-specific mortality and two major OSCC risk factors (cigarette smoking and alcohol use). The genome-wide hierarchical clustering on CNA/LOH events did not reveal associations with tumor characteristics or mortality (S3 Table, S2 Fig for CNA; S3 Fig for LOH). However, LOH clusters showed a statistically significant difference in smoking status (S3 Table, Fisher Exact test p = 0.0069).

### Association of CNA/LOH at specific genome regions with tumor characteristics and OSCC-specific mortality

**Fig 3:**
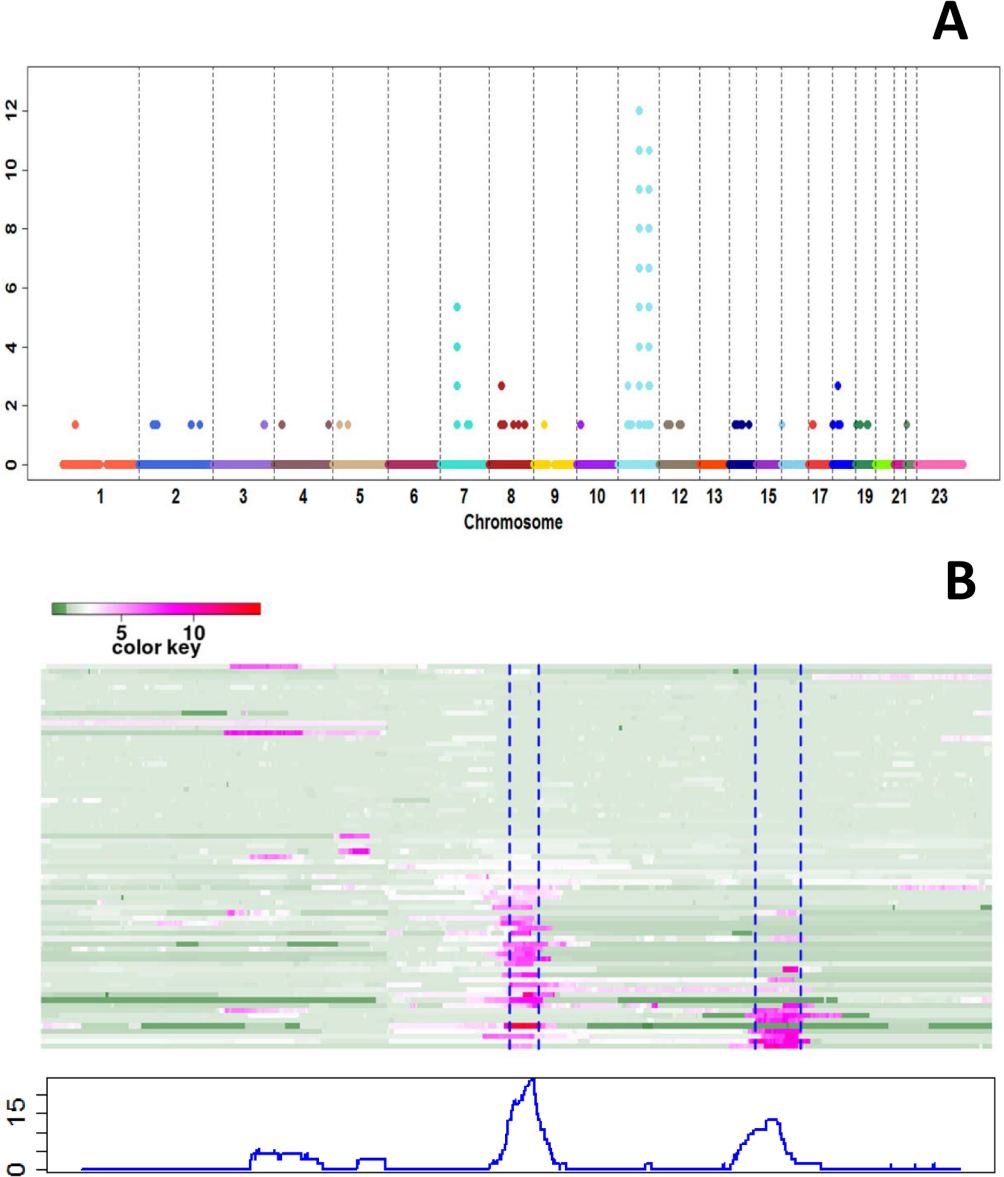
Results on copy number amplification. A) High-level copy number amplifications. Each dot represents one probe. *X-axis*: genome order of probes from 22 autosomes. *Y-axis*: Percentage of samples showing high copy number amplification at each probe. High copy number amplification is defined as the difference between copy number in a tumor sample vs. that in the corresponding blood sample being greater than or equal to 5. B) Chromosome 11 tumor copy number data. The top panel shows the heatmap of tumor CN data with probes in columns and 75 subjects in rows. The blue dashed lines label the high-level amplification regions 1 and 2. The bottom panel shows the percentage of samples having high-level copy number amplifications (CNA> = 5) at each probe.

**Fig 4:**
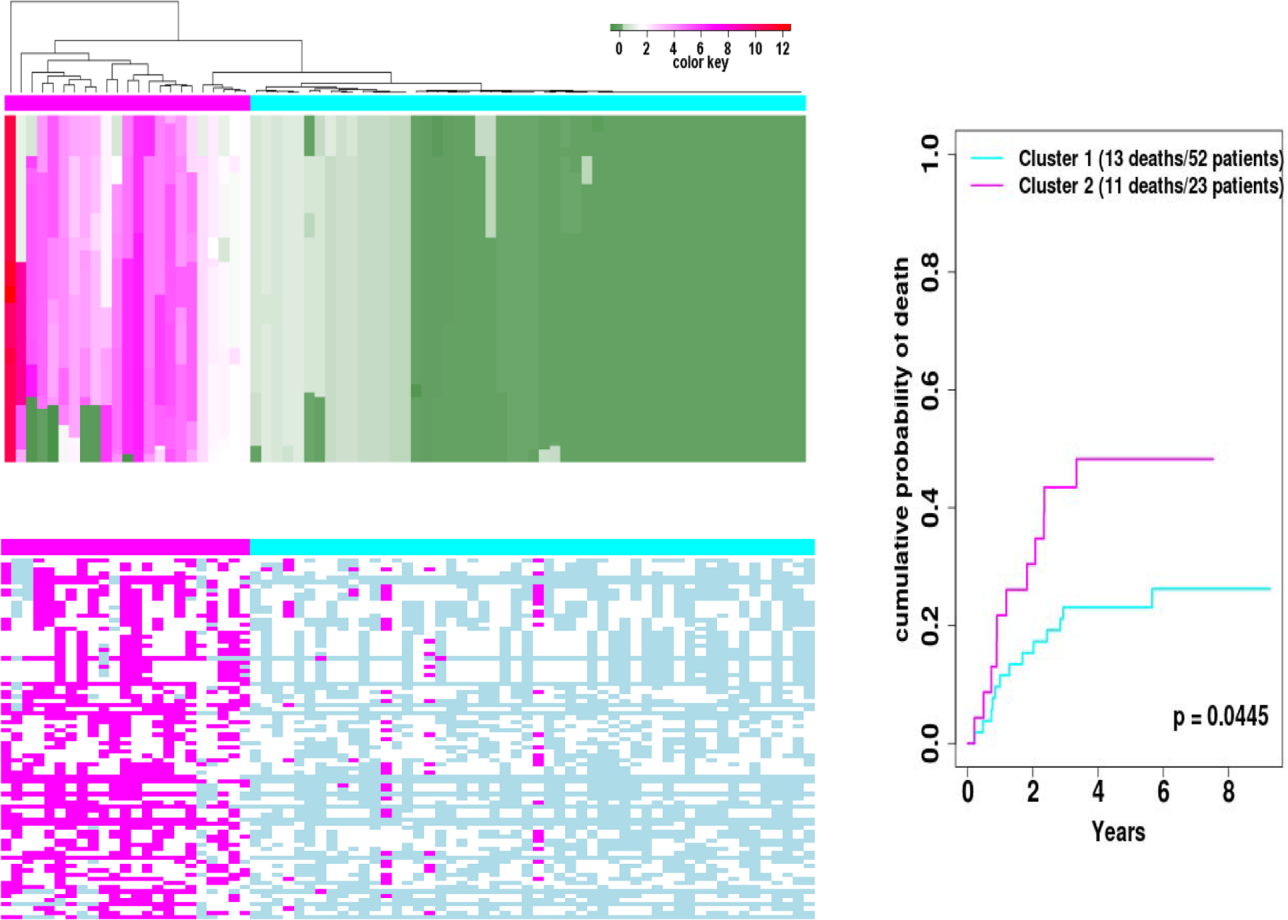
CNA and LOH data for the high-level amplification region 1 in 11q 13.1–14.3 (85 probes from nucleotide position nt68683098 to nt70375682). The top left panel shows the heatmap of tumor CNA data, and the color key indicates the estimated copy number. The bottom left panel shows the heatmap of LOH data (magenta, LOH present; blue, no LOH; white, not informative). In both heatmaps, the rows stand for SNPs and the columns stand for samples. Based on the CNV data, we clustered the samples into two groups (23 patients in the magenta group and 52 patients in the blue group) using hierarchical clustering algorithm. The right panel shows the cumulative incidence curves of the OSCC-specific death of the patients in the two clusters. The X-axis indicates the years between surgery and last follow-up or death. The Y-axis indicates the cumulative incidence of death due to OSCC. OSCC-specific mortality of the two clusters of patients was significantly different (p = 0.0445) according to log rank test.

For CNA, we focused on regions with high-level copy number amplification (defined as the difference between estimated copy number in the tumor sample vs. that in the matched blood sample being ≥5) (Fig 3A). Two regions on Chr. 11 (Fig 3B) have frequent high-level copy number amplification; region 1: 11q13.1–14.3, nt68683098 to nt70375682, with CNA≥5 observed in *MYEOV*, *CCND1*, *ORAOV1*, *FGF19*, *FGF4*, *FGF3*, *ANO1*, *FADD*, *PPF1A1*, *CTTN* and *SHANK2*; and region 2: 11q22-24, nt99440128 to nt102804260, with CNA≥5 observed in MMP-1, -3, -7, -8, -10, -12, -13, -20, and -27; *ARHGAP42*; *YAP1*; *TRPC6*; *CNTN5*; *DYNC2H1*; *TMEM123*; *WTAPP1*; *LOC10105*; *BIRC3*; *KIAA1377*; *C11orf70*; *DCUN1D5*; *PGR*; and *ANGPTL5*. The percentages of samples showing high-level CN amplification in these two regions are approximately 20% and 10%, respectively (Fig 3B). Heatmaps illustrating the LOH and CNA data of these two regions are shown in Fig 4 and S4 Fig, respectively. Interestingly, while 8–29% of the study participants have LOH in region 1, most of the copy number gain events in region 1 are also accompanied with LOH (Fig 4). Applying hierarchical clustering on CNA data in this region further divided the patients into two major groups: 23 patients with numerous copy-number-gain-LOH events; and 52 with few LOH or CNA events (Fig 4 left top panel). Cumulative incidence analyses indicate that the group of 23 patients had a greater likelihood of OSCC-specific mortality (log rank test p = 0.0445 comparing 11 deaths of 23 patients vs. 13 deaths of 52 patients, Fig 4, right panel). Patients in these two groups do not appear to differ by TNM staging, tumor site and history of tobacco smoking and alcohol use (S4 Table). When the patients were clustered into two groups based on their LOH events in this region, the cumulative incidence curves did not show a statistically significant difference between the two groups (data not shown). When hierarchical clustering on CNA data in region 2 subdivided the patients into two subgroups, no statistically significant difference in OSCC-specific mortality between the subgroups was observed (S4 Fig). There was a small suggestion that CNA events in this region might be related to history of tobacco use (S4 Table, Fisher Exact test p = 0.067). There were greater percentages of smokers, especially current smokers, than non-smokers in cluster 1, which had fewer CNA events than cluster 2; the percentages of current, former and never smokers were 48%, 36% and 16%, respectively. Current and former smokers were less likely than non-smokers to be in cluster 2, which had greater number of CNA events; the respective percentages of current, former and never smokers were 23%, 31% and 46%. Thus, these results suggest that smokers are less likely to have abnormal CNA in region 2.

For LOH, we focused on Chr. 9p (Fig 2), which has the most frequent *large-region-LOH* in this data set. The two clusters of patients defined by LOH frequencies on this chromosome arm, with cluster 2 harboring more LOH events, differed significantly in terms of their smoking history (S5 Table, p = 0.002). Patients in cluster 2 were more likely to be smokers, especially current smokers; the respective percentages were 57%, 29% and 14% for current, former and never smokers. These results suggest that tobacco exposure may result in a large number of LOH events on Chr. 9p. No statistically significant differences were detected between two groups’ cumulative probability of OSCC-specific mortality (S5 Fig, p = 0.782).

### Association of individual SNPs with OSCC-specific mortality

**Fig 5:**
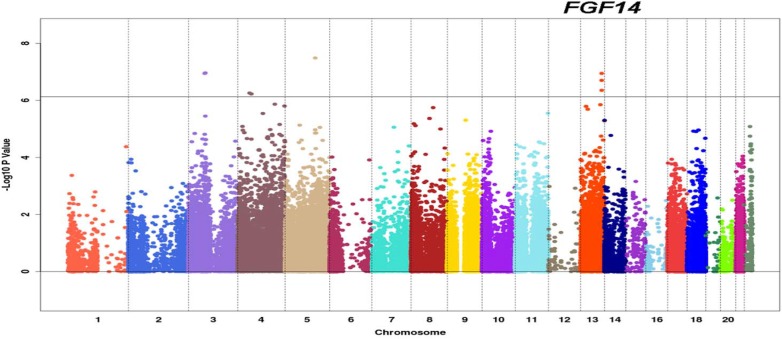
Manhattan plot of p-values for testing interaction effects between each probe’s LOH status and its DNA copy number in Cox regression models for OSCC-specific survival. The X-axis shows the genome order with different chromosomes separated by vertical dashed grey lines. The Y-axis shows the negative log_10_ p-values from log-likelihood ratio test for testing the interaction term (CNV*LOH) in the Cox model that included terms for sex, age, and smoking history. The horizontal grey line corresponds to Bonferroni cutoff 0.05/66875.

**Fig 6:**
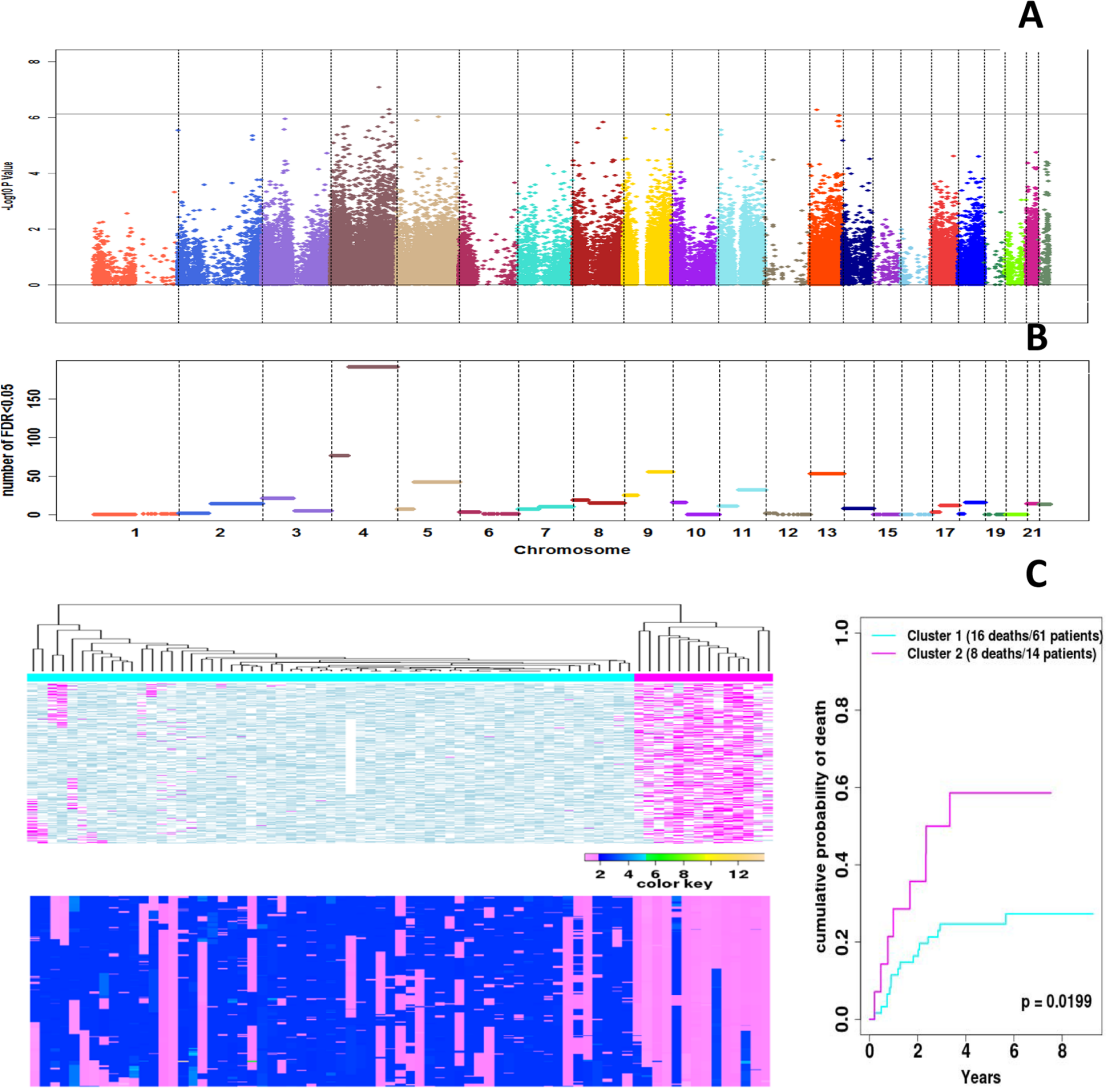
Association of each probe’s LOH status and DNA copy number with OSCC-specific mortality. A) Manhattan plot of p-values for testing the association of each probe’s LOH status and DNA copy number with OSCC-specific mortality. The X-axis shows the genome order with different chromosomes separated by vertical grey lines. The Y-axis shows the negative log_10_ p-values from likelihood ratio test for testing the main effect and interaction terms for CNV & LOH in the Cox model that also include terms for sex, age, and smoking history. The horizontal grey line corresponds to Bonferroni cutoff 0.05/66875. B) Number of loci in each chromosome arm, whose p-value survived the FDR 0.05 cutoff in A). The X-axis again stands for the genome order. The Y-axis stands for the number of probes (probes from the same chromosome arm share the same value). C) LOH and CNA data for the whole Chr. 4q (8975 probes from nucleotide position from 63637813 to 183550869). The top left panel shows the heatmap of LOH data (magenta, LOH present; blue, no LOH; white, not informative). The bottom left panel shows the heatmap of tumor CN data, and the color key indicates the copy number. In both heatmaps, the rows stand for SNPs and the columns stand for samples. Based on the LOH data, we clustered the samples into two groups (14 patients in the magenta group and 61 patients in the blue group) using hierarchical clustering algorithm. The right panel shows the Cumulative Incidence curves of the OSCC-specific death of the patients in the two clusters. The X-axis indicates the years between surgery and last follow-up or death. And the Y-axis indicates the mortality rate. Survival of the two clusters of patients was significantly different (p = 0.0199) according to log rank test.

Cox regression models were fit to assess the association between OSCC-specific mortality and CNA or LOH of each probe, as well as their interaction, adjusting for sex, age and smoking. After filtering out probes with LOH events occurring in fewer than 5 samples, there were 66,875 probes considered in the analysis. The Manhattan plots in Fig 5 and Fig 6A show the-log_10_(p value) from the likelihood ratio tests for the interaction effect between CNA and LOH of each SNP, and the overall contribution of each SNP’s CNA and LOH information in the Cox regression, respectively. There were eight probes for which the interactions between CNAs and LOHs appear to be statistically significant in their Cox models based on Bonferroni corrected significance level of 0.05 (p-value <7.48e-07) (Fig 5, Table 1). Among the eight probes, three have complimentary sequence to *FGF14*. The corresponding results shown in Table 1 suggest an interesting modifying effect of LOH on the association between CNA of *FGF14* and mortality in that copy number gains of this gene increase the hazard rate in those patients with LOH at this gene (positive lnHR), while decrease the hazard rate in those patients without LOH (negative lnHR).

**Table 1:** Eight probes with statistically significant interaction effects between CNA and LOH on OSCC-specific mortality.

Cyto-band	SNP ID	RS position	Gene Symbol	Nucleotide position	p.all^ⱡ^	p.CNA*LOH^ⱡⱡ^	Direction of CNA association	
							**No LOH**	**LOH present**
3p26	SNP_A-2231666	rs4478080	FAM19A1	68320588	1.12E-06	1.09E-07	*	+
3p26	SNP_A-8591805	rs2060023	MAGI1	65974572	2.71E-06	1.13E-07	*	+
4q12	SNP_A-1855888	rs17750221	—	55040244	9.91E-06	5.95E-07	+	-
4p12	SNP_A-2196903	rs7694862	TEC	48190372	2.11E-06	5.54E-07	*	-
5q35	SNP_A-1806221	rs4343835	SNX2	122116630	9.44E-07	3.27E-08	-	+
13q34	SNP_A-2182075	rs951348	FGF14	102788188	2.08E-06	4.44E-07	*	+
13q34	SNP_A-4289264	rs952645	FGF14	102787731	8.74E-07	1.13E-07	-	+
13q34	SNP_A-8316408	rs7994713	FGF14	102789615	1.40E-06	1.99E-07	-	+

‡ p-values from the log-likelihood ratio test for terms for CNA, LOH, and the multiplicative interaction of CNA and LOH (LOH*CAN) in the Cox regression model.

‡‡ p-values from the log-likelihood ratio tests of the interaction term (cnv*loh).

* Direction of association undetermined.

For each SNP, we tested the joint effect of LOH, CNA and their interaction term in the Cox models. We found four probes (with one each corresponding to *PALLD*, *DDX60L* and *MAML3*) significantly associated with OSCC-specific mortality with the Bonferroni corrected p-values <0.05 (Fig 6A, Table 2). Results in Fig 6A also suggest that there are a large number of probes on Chr. 4 showing moderate association (low p-values). Indeed, at FDR = 0.05, we find 675 SNPs showing significant association with OSCC deaths. Among these, a large percentage (28.3%) is on Chr. 4q (Fig 6B). This motivated us to assess the connection between LOH/CNA of this chromosome arm and mortality. We clustered patients into two groups (14 vs. 61) based on LOH on 4q. Patients in the two clusters were similar in tumor size, site, nodal status, smoking and alcohol use history, but differ by AJCC stage (S6 Table, Fisher Exact test p = 0.0477). Cumulative incidence curves of OSCC-specific mortality of these two groups showed a statistically significant difference (Fig 6C, log rank test p = 0.0199). Furthermore, the two LOH-based clusters show significantly different association with patients’ mortality even when AJCC stage was included in the adjustment (log-likelihood ratio test p = 0.00161). We conducted the same analysis using CNA data of 4q, but didn’t detect a significant difference in their association with OSCC-specific mortality (S6 Fig).

**Table 2:** List of 4 probes of whom the combined information of CNA and LOH are significant for predicting OSCC-specific m.

Cyto-band	SNP ID	RS position	Gene Symbol	Nucleotide position	P (CNA, LOH, & CNA*LOH terms)l‡	P (CNA*LOH Interaction)‡‡	Direction of CNA association	
							**No LOH**	**LOH present**
4q33	SNP_9242309	rs12647997	PALLD	169663775	7.47E-07	0.63	*	+
4q33	SNP_A-8393999	rs9685229	DDX60L	169371116	5.26E-07	1.54E-05	+	+
4q31.1	SNP_A-8706233	rs11100449	MAML3	141171560	8.53E-08	4.15E-03	*	+
13q14	SNP_A-2115691	rs7339301	—	39637333	5.42E-07	1.62E-06	-	+

‡ p-values from the log-likelihood ratio tests for the combined term (cnv+loh+cnv:loh) in the cox regression model “OSCC-specific survival ~ CNV + LOH + CNV:LOH + sex + age +smoking history”.

‡‡ p-values from the log-likelihood ratio tests of the interaction term (cnv:loh).

* Direction of association undetermined.

## Discussion

This study used LCM isolated tumor cells from fresh frozen tumor samples and paired peripheral blood leukocytes and the high density Affymetrix Genome-wide Human SNP Array 6.0 to interrogate LOH/CNA events in HPV-negative OSCC and their association with OSCC-specific mortality. It detected a wide array of LOH and CNA events among tumors from 75 patients. All tumors harbored LOH and/or CNA, with no two exhibiting the same LOH/CNA patterns on the genome-wide level.

This study confirms and adds to previous evidence regarding LOH and CNA in certain chromosomal regions and gene loci that may play an important role in oral cancer biology and prognosis. The observation of frequent LOH events on Chr. 3p, 9p, 11q and 17p is consistent with previous reports [6, 36–61]. A number of genes on Chr. 3p have been proposed to be HNSCC TSG that are shared with breast or colon cancer [62]. Based on the respective top 100 SNPs exhibiting the most frequent LOH or copy number loss in our study, the potential TSG on Chr. 3p would include *TGFBR2*, *CNTN4*, and *CHL1* (reported for colorectal cancer) and *CNTN6* (for breast cancer [63]), as well as *FHIT*, *ROBO1-GRE1* and *SETMAR-LRRN1*. So far, few studies have proposed HNSCC TSG in Chr. 9p [44]. Our results on extremely frequent LOH (in >80% of patients tested) suggest that eight genes (*FLJ35024*, *KDM4C*, *CCDC171*, *IL33*, *PTPRD*, *TTC39B*, *SH3GL2*, *FREM1*) within Chr. 9p21.2–24.3 may be TSG for HNSCC that, with the exception of *PTPRD and SH3GL2*, have not been recognized previously. *KDMC4* is a histone demethylase involved in chromatin remodeling [64, 65]. IL33 expression has been reported to be elevated in HNSCC and may promote cell migration and invasion [66]. *PTPRD* encodes for a phosphatase that regulates cell cycle. Its inactivation via homozygous deletion and/or mutation has been reported in several cancers, including HNSCC [67–69]. Our finding adds to the reported mechanisms of its inactivation. Variant genotypes of *TTC39B* have been associated with dyslipidemia [70, 71]. The present study is the first to implicate it in cancer and raises the question of what role lipid metabolism may play in OSCC pathophysiology. There is some prior evidence to support *SH3GL2* as a HNSCC TSG in that its decreased expression and/or deletion was associated with laryngeal cancer [72, 73]. The current study is the first to suggest its potential role as a TSG in OSCC, and that LOH may be another mechanism for its dysregulation. *FREM-1* encodes for an extracellular matrix adhesion protein [74]. The current study is the first for its implication in cancer. Further human and model system studies to verify our new findings implicating *KDM4C*, *PTPRD*, *TTC39B*, *SH3GL2*, *FREM1* as potential TSG are warranted. It is noteworthy that at least for the top 100 SNPs associated with the most frequent LOH, the LOH events were accompanied by copy number gain, loss, or neither gain nor loss, attesting to the complexity of genomic changes in OSCC. It is also notable is that the top 100 SNPs with copy number gains are all in 8q24. The genes harboring these SNPs (S1B Table) could be potential oncogenes for OSCC and warrant further investigation.

Our observed DNA copy number gains of oncogenes *EGFR* and *ERBB2* and copy number loss of TSG *CDKN2A* and *SMAD4* are consistent with prior reports on HNSCC [6, 75–84]. *FAT1* encodes for a cadherin protein implicated in the adhesion and migration of oral cancer cells [85]. Results from the Cancer Genome Atlas project [86] and the India Project conducted by the International Cancer Genome Consortium (India project team of the International Cancer Genome Consortium, 2013) showed that *FAT1* is mutated in 20%-40% of HNSCC. Homozygous loss of *FAT1* has been reported in a study of oral cancer [87]. Based on its frequent mutations and copy number loss in cancer, *FAT1* has been hypothesized as a TSG. Our results showing copy number loss in *FAT1* adds support to this hypothesis.

Amplification of 11q13.1–14.3 region in HNSCC has been observed by others [6, 84, 88–96]. Our observation of high-level copy number amplification of *MYEOV*, *CCND1*, *ORAOV1*, *FGF19*, *FGF4*, *FGF3*, *ANO1*, *FADD*, *PPF1A1*, *CTTN* and *SHANK2* in this region is consistent with prior reports and supports the hypothesis that they might be oncogenes and warrants further investigation into their potential as therapeutic targets. Our observation that the majority of patients who harbor high copy number amplification in this region also have LOH events and that patients with copy-number-gain-LOH in this region have poorer survival than those who rarely have CNA or LOH in this region represents a new discovery and requires further validation. The fact that patients clustered by CNA rather than LOH showed a significant difference in OSCC-specific mortality underscores the importance of gene amplification in this region and the need to investigate the amplified genes in their role as potential drivers for OSCC aggressiveness, prognostic indicators, and targets for therapeutic intervention.

Our results showed that region 2 of Chr. 11q (11q22-24) also contains many genes with high level of amplification (CNA≥5), including many MMPs (MMP-1, -3, -7, -8, -10, -12, -13, -20, and -27). This observation underscores the pivotal function of metalloproteinases in OSCC progression. High level of amplification of *MMP-1*, *-3*, *-7*,*-10*,*-12*, *-13* in HNSCC has previously been reported [97], while amplification of *MMP-8*, *-20*, and *-27* has not. *ARHGAP42* is a RhoGTPase activating protein. No prior reports linking it to cancer. Others have hypothesized *YAP1* to be a driver gene for HNSCC [98–100]; our observed high level *YAP1* amplification lends support to this hypothesis. High *TRPC6* expression has been observed in other cancers [101–105] and was reported to be associated with poor prognosis of esophageal cancer [105]. *CNTN5* encodes an adhesion molecule that mediates cell surface interactions during neural development. Our finding is the first to link *CNTN5* with cancer. Whether it plays a role in perineural invasion by participating in reciprocal signaling between nerves and OSCC as demonstrated by plexins and semaphorins in HNSCC models [106] warrants examination. *DYNC2H1* encodes a dynein protein purported to be involved in immune system and cytoskeleton remodeling. *TMEM123* encodes a transmembrane maturation marker for dendritic cells in mice [107]. *WTAPP1*, a pseudogene with unknown function, has been reported to be associated with Wilms’ tumor. Amplification of *BIRC3* has been reported in acute myeloid leukemia [108] and pancreatic cancer [109]. Little is known about *KIAA1377*. *DCUN1D5* encodes for a protein involved in neddylation of cullin. Its overexpression was reported in laryngeal cancer [110]. *ANGPTL5* encodes for angiopoietin-like 5 protein that supports the engraftment of human hematopoietic stem cells in NOD-SCID mice [111]. Our results showing high copy number of this gene in OSCC is the first to implicate its role in carcinogenesis.

We observed that copy number gains of *FGF14* are associated with an increased risk of OSCC-specific mortality in patients with LOH in this gene, and a decreased risk in patients without LOH. This observed interaction effect between LOH and CNA suggests that the two alleles might involve very different functional consequences. Such phenomenon can be detected only when both LOH and CNA information are available–a strength of our study. While interesting, these findings need to be interpreted with caution and validated in larger populations because the sample size of this study is still quite limited. Dysregulation of FGF signaling in cancer and FGFs’ potential as therapeutic targets are active areas of research [112–115]. While having sequence homology to FGFs, FGF14 does not activate FGF receptor as a true ligand [116]. Instead, it was found to be an intracellular modulator of voltage-gated sodium channel essential for regulating neuronal activities [117]. It would be of interest to examine the biological function of FGF14 in oral carcinogenesis and whether its expression is in anyway related to perineural invasion of OSCC.

Our results showed that CNA, LOH, and their interaction for a probe in each of *PALLD*, *DDX60L* and *MAML3* genes were significantly associated with OSCC mortality. *PALLD encodes* palladin, an actin-associated protein essential for the regulation of cell morphology and motility. Its upregulation has been reported in pancreatic cancer [118, 119] and, was associated with poor survival among patients with renal cell carcinoma [120]. Our observation that copy number gain LOH in *PALLD* is associated with particularly poor survival is consistent with the observation in pancreatic cancer and with our prior report of an association between upregulation of alpha-actinin, another player in cytoskeleton remodeling, and poor progression-free survival among OSCC patients [121]. *DDX60L* encodes for DEAD protein with unknown function. The protein encoded by *MAML3* has been shown to be essential for Notch signaling *in vivo* [122]. Notch signaling is utilized effectively in numerous cellular and developmental processes through its multiplicity of receptors (Notch 1–4) and ligands including MAML1, MAML2 and MAML3 that serve as coactivators [123]. Notch 1 has been shown to be frequently mutated in HNSCC with nearly 40% of the 28 mutations identified predicting a truncated gene product [124]. The authors of that paper suggested that *NOTCH1* may function as a TSG rather than an oncogene in HNSCC. Our result showing having LOH in *MAML3* was associated with poor survival even in the context of copy number gains lends support to this hypothesis

Heterozygosity and partial or complete loss of Chr. 4q have been reported for verrucous hyperplasia/carcinoma [125] and HNSCC [126, 127]. Unlike the present study, that earlier report [126] on HNSCC did not find LOH on Chr. 4q to be associated with survival. While the reason for the difference is unknown, it might have to do with the fact that the earlier report was based on the examination of 33 polymorphic microsatellites while our study involved the examination of 189 LOH events.

Thus, even with a limited number of 75 patients, the current study has confirmed a number of prior findings on LOH and CNA in oral cancer as well as discovered a number of chromosomal regions that contain potential oncogenes and TSG for OSCC that warrant further investigation. Results of the current study also point to the heterogeneity and complexity of genomic alterations in OSCC and the need to take this into account when designing therapeutic interventions for oral cancer patients. Larger studies are needed to confirm or refute the observed associations of LOH, CNA, or their interactions with OSCC outcomes, and, if confirmed, to examine the clinical utility of these findings. Conducting larger studies could also discover new somatically-altered chromosomal regions that could not be detected with the multiple comparison penalties in our current study.

## Supporting Information

S1 Fig Percentage of probes showing CNA and LOH events in each OSCC. The top three plots show information for CNA normal, gain and loss. The bottom plot shows the LOH events. The 75 OSCC are sorted by the percentages of probes showing LOH events.
(DOCX)

S2 Fig Cumulative incidence curves of the OSCC-specific death for patients clustered by CNA. The left panel shows the heatmap of CNA segments across all 22 autosomes (separated by the blue dotted lines). Rows are individuals and columns are probe segments. Two patient groups resulting from hierarchical clustering are labelled with purple and blue in the color bar on the left side of the heatmap. The right panel shows the cumulative incidence curves of the OSCC-specific death for the patients in each of the two clusters. The X-axis indicates the years between surgery and last follow-up or death due to OSCC The Y-axis indicates the mortality rate.
(DOCX)

S3 Fig Cumulative Incidence curves of OSCC-specific death of patients clustered by LOH. The left panel shows the heatmap of LOH across all 22 autosomes (separated by the blue dotted lines; magenta, LOH present; blue, no LOH; white, not informative). Rows are individuals and columns are probe segments. Two patient groups resulted from hierarchical clustering are labelled with magenta and blue in the color bar to the left of the heatmap. The right panel shows the Cumulative Incidence curves of OSCC-specific death of the patients in the two clusters. The X-axis indicates the years between surgery and last follow-up or death. The Y-axis indicates the mortality rate.
(DOCX)

S4 Fig CNA and LOH data for the high-level amplification region 2 on Chr. 11 (115 probes from 11q22-24 with nucleotide position from nt99440128 to nt102804260). The top left panel shows tumor CNA data, and the color key indicates the number of copies. The bottom left panel shows the heatmap of the heatmap of LOH data (magenta, LOH present; blue, no LOH; white, not informative). In both heatmaps, the rows stand for SNPs and the columns stand for samples. Based on the CNV data, we cluster the samples into two groups (13 patients in the red group and 62 patients in the blue group) using hierarchical clustering algorithm. The right panel shows the Cumulative Incidence curves of the OSCC-specific death of the patients in the two clusters. The X-axis indicates the years between surgery and last follow-up or death. And the Y-axis indicates the mortality rate.
(DOCX)

S5 Fig LOH and CNA data for Chr. 9p with frequent LOH event (2579 probes from nucleotide position from 36587 to 38761831). The top left panel shows the heatmap of LOH data (magenta, LOH present; blue, no LOH; white, not informative). The bottom left panel shows the heatmap of tumor CNA data, and the color key indicates the copy number. In both heatmaps, the rows stand for SNPs and the columns stand for samples. Based on the LOH data, we clustered the samples into two groups (24 patients in the magenta group and 51 patients in the blue group) using hierarchical clustering algorithm. The right panel shows the Cumulative Incidence curves of the OSCC-specific death of the patients in the two clusters. The X-axis indicates the years between surgery and last follow-up or death. The Y-axis indicates the mortality rate.
(DOCX)

S6 Fig CNA and LOH data for the whole Chr. 4q region (8975 probes from nucleotide position from 63637813 to 183550869). The top left panel shows the heatmap of tumor CN data, and the color key indicates the copy number. The bottom left panel shows the heatmap of LOH data (magenta, LOH present; blue, no LOH; white, not informative). In both heatmaps, the rows stand for SNPs and the columns stand for samples. Based on the CNA data, we clustered the samples into two groups (15 patients in the magenta group and 60 patients in the blue group) using hierarchical clustering algorithm. The right panel shows the Cumulative Incidence curves of the OSCC-specific death of the patients in the two clusters. The X-axis indicates the years between surgery and last follow-up or death. The Y-axis indicates the mortality rate. Survival of the two clusters of patients was significantly different (p = 0.199) according to log rank test.
(DOCX)

S1 Table Selected characteristics of HPV-negative oral squamous cell cancer patients, University of Washington Affiliated Institutions, 2004–2010 (n = 75). (DOCX)

S2 Table Top 100 most frequent LOH, CN gain and CN loss. (XLSX)

S3 Table Selected characteristics of OSCC patients according to clusters defined by genome-wide tumor CNA or LOH events, University of Washington Affiliated Institutions, 2004–2010. (DOCX)

S4 Table Selected characteristics for patients in clusters defined by CNA in Chr. 11, Region 1 (11q13.1–14.3, nt 68683098-nt 70375682) and Region 2 (11q22-24, nt99440128-nt102804260). (DOCX)

S5 Table Selected characteristics of patients in clusters defined by LOH on Chr. 9p. (DOCX)

S6 Table Selected characteristics for patients in clusters defined by LOH on Chr. 4q. (DOCX)
