# Supplementary material for: Genome-Wide Loss of Heterozygosity and DNA Copy Number Aberration in HPV-Negative Oral Squamous Cell Carcinoma and Their Associations with Disease-Specific Survival
Source: PLoS One. 2015 Aug 6;10(8):e0135074. doi: 10.1371/journal.pone.0135074 (PMC4527746; doi:10.1371/journal.pone.0135074)
Supplement: S1 Fig — The top three plots show information for CNA normal, gain and loss. The bottom plot shows the LOH events. The 75 OSCC are sorted by the percentages of probes showing LOH events. (DOCX) [file pone.0135074.s001.docx]

**
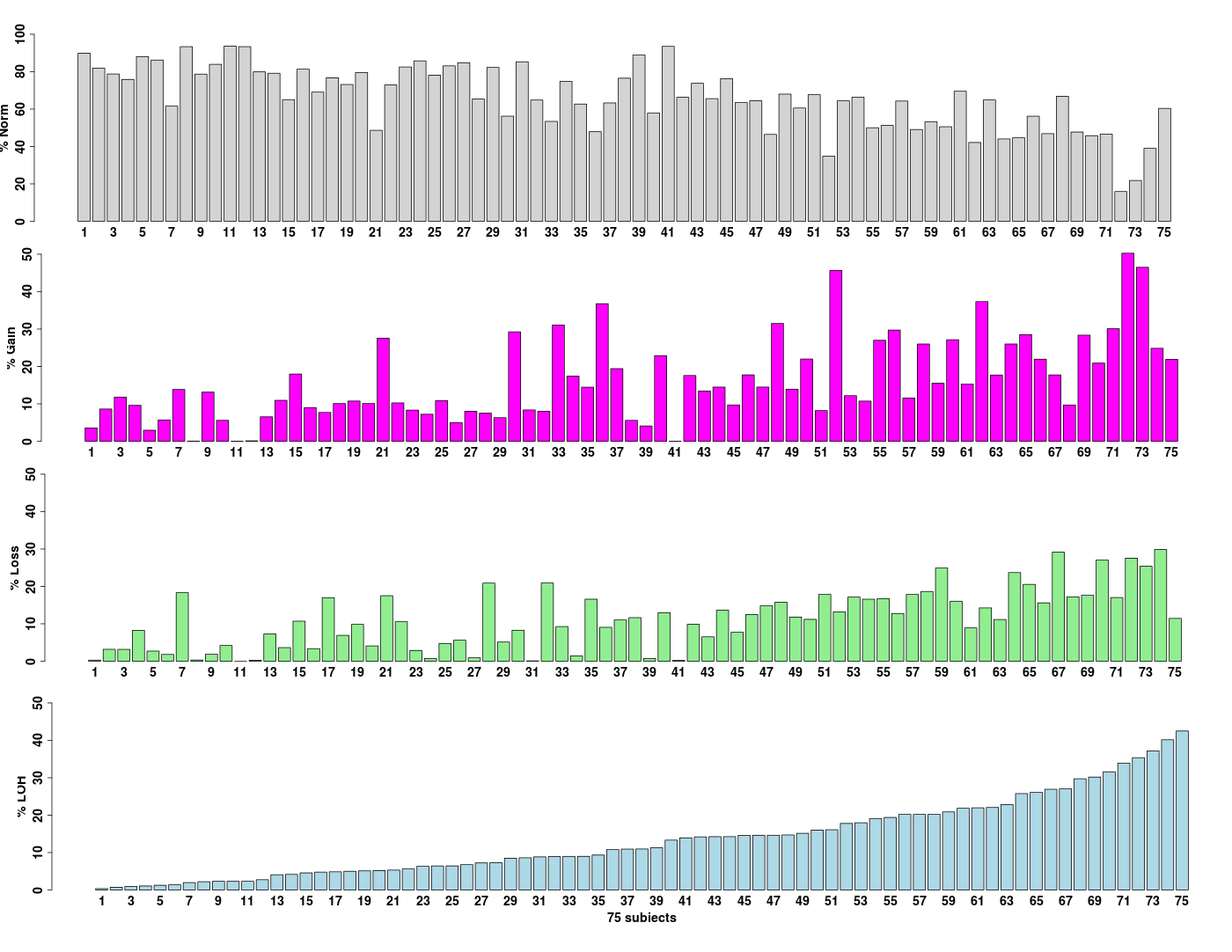
**

**Figure S1:** Percentage of probes showing CNA and LOH events in each OSCC. The top three plots show information for CNA normal, gain and loss. The bottom plot shows the LOH events. The 75 OSCC are sorted by the percentages of probes showing LOH events.
