# Supplementary material for: Genome-Wide Loss of Heterozygosity and DNA Copy Number Aberration in HPV-Negative Oral Squamous Cell Carcinoma and Their Associations with Disease-Specific Survival
Source: PLoS One. 2015 Aug 6;10(8):e0135074. doi: 10.1371/journal.pone.0135074 (PMC4527746; doi:10.1371/journal.pone.0135074)
Supplement: S2 Fig — The left panel shows the heatmap of CNA segments across all 22 autosomes (separated by the blue dotted lines). Rows are individuals and columns are probe segments. Two patient groups resulting from hierarchical clustering are labelled with purple and blue in the color bar on the left side of the heatmap. The right panel shows the cumulative incidence curves of the OSCC-specific death for the patients in each of the two clusters. The X-axis indicates the years between surgery and last follow-up or death due to OSCC The Y-axis indicates the mortality rate. (DOCX) [file pone.0135074.s002.docx]

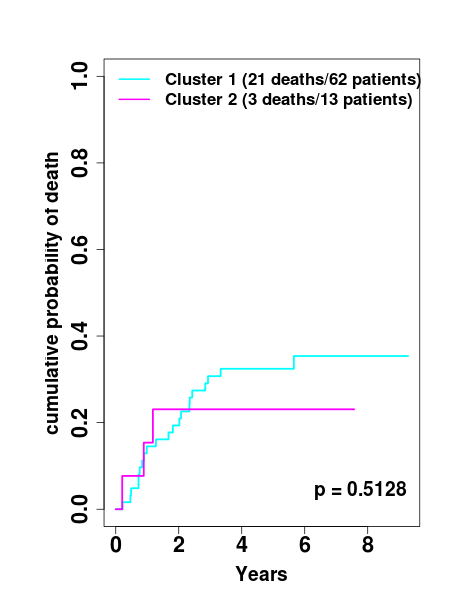

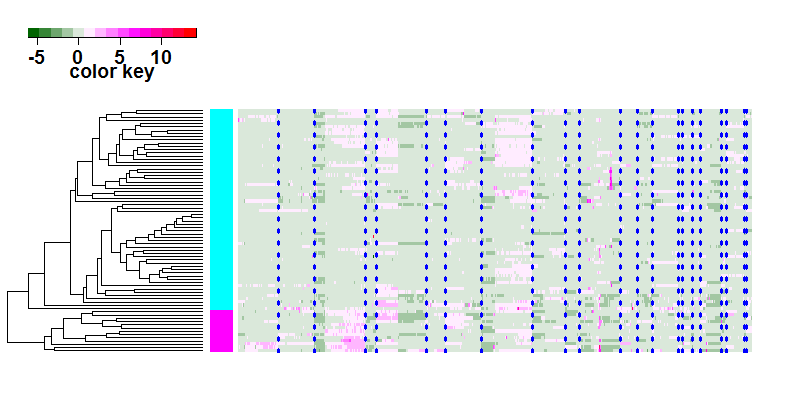


**Figure S2:** **The left panel** shows the heatmap of **CNA** segments across all 22 autosomes (separated by the blue dotted lines). Rows are individuals and columns are probe segments. Two patient groups resulting from hierarchical clustering are labelled with purple and blue in the color bar on the left side of the heatmap. **The right panel** shows the cumulative incidence curves of the OSCC-specific death for the patients in each of the two clusters. The X-axis indicates the years between surgery and last follow-up or death due to OSCC The Y-axis indicates the mortality rate.
