# Supplementary material for: Genome-Wide Loss of Heterozygosity and DNA Copy Number Aberration in HPV-Negative Oral Squamous Cell Carcinoma and Their Associations with Disease-Specific Survival
Source: PLoS One. 2015 Aug 6;10(8):e0135074. doi: 10.1371/journal.pone.0135074 (PMC4527746; doi:10.1371/journal.pone.0135074)
Supplement: S3 Fig — The left panel shows the heatmap of LOH across all 22 autosomes (separated by the blue dotted lines; magenta, LOH present; blue, no LOH; white, not informative). Rows are individuals and columns are probe segments. Two patient groups resulted from hierarchical clustering are labelled with magenta and blue in the color bar to the left of the heatmap. The right panel shows the Cumulative Incidence curves of OSCC-specific death of the patients in the two clusters. The X-axis indicates the years between surgery and last follow-up or death. The Y-axis indicates the mortality rate. (DOCX) [file pone.0135074.s003.docx]

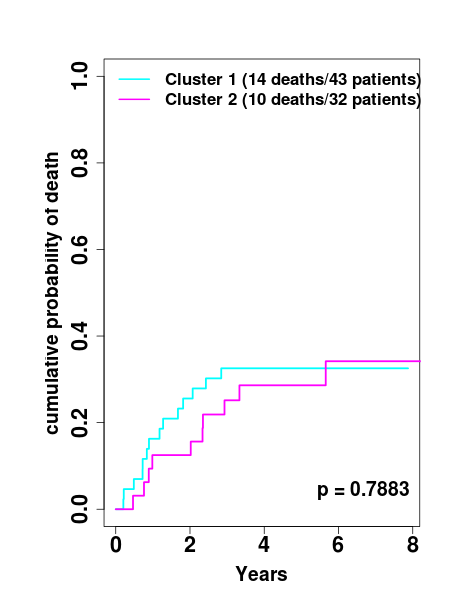

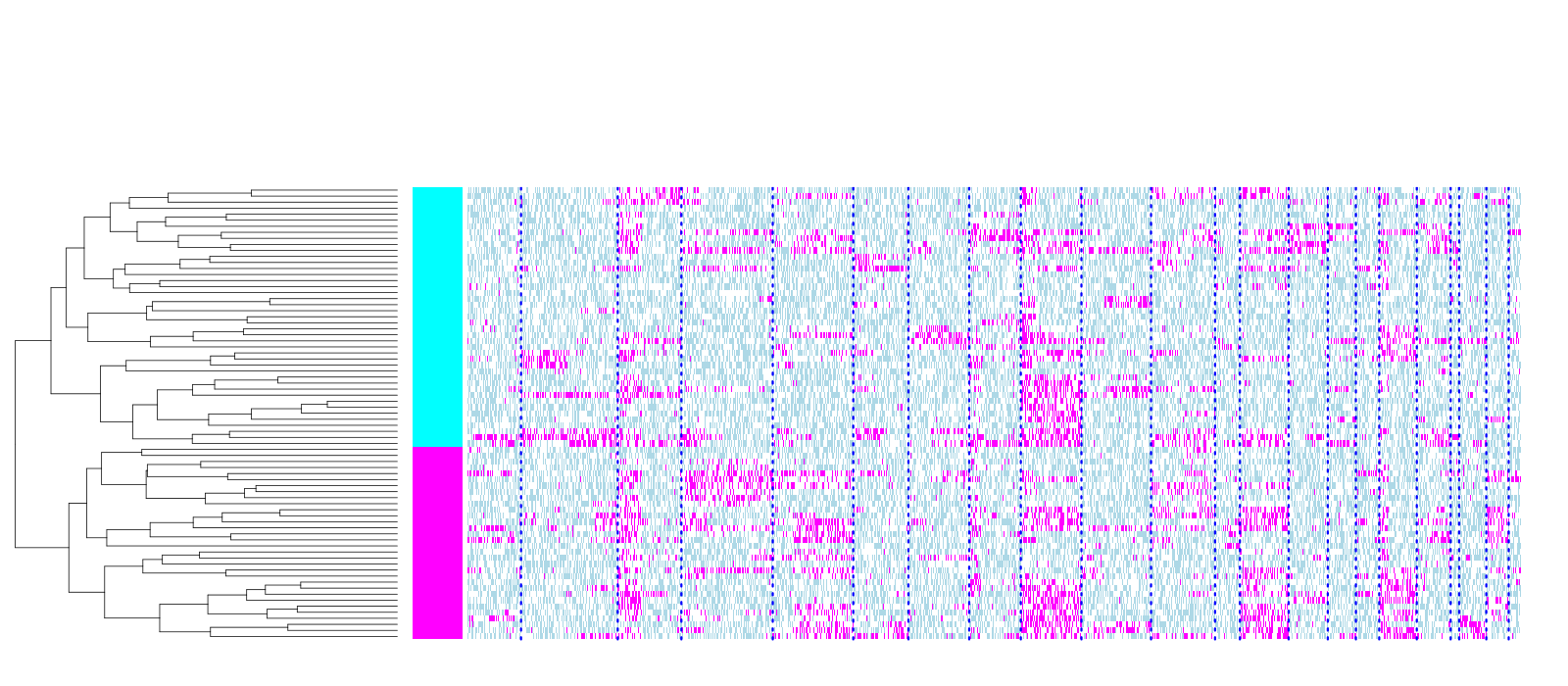


**Figure S3:** **The left panel** shows the heatmap of **LOH** across all 22 autosomes (separated by the blue dotted lines; magenta, LOH present; blue, no LOH; white, not informative). Rows are individuals and columns are probe segments. Two patient groups resulted from hierarchical clustering are labelled with magenta and blue in the color bar to the left of the heatmap. **The right panel** shows the Cumulative Incidence curves of OSCC-specific death of the patients in the two clusters. The X-axis indicates the years between surgery and last follow-up or death. The Y-axis indicates the mortality rate.

Heatmap of LOH in segment level cross all 22 autosomes (separated by the blue lines). Red is LOH 1 blue is LOH 0, white is missing; Rows are individuals and columns are probe segments. The row slide colors red/blue are the two hierarchical clusters.
