# Supplementary material for: Genome-Wide Loss of Heterozygosity and DNA Copy Number Aberration in HPV-Negative Oral Squamous Cell Carcinoma and Their Associations with Disease-Specific Survival
Source: PLoS One. 2015 Aug 6;10(8):e0135074. doi: 10.1371/journal.pone.0135074 (PMC4527746; doi:10.1371/journal.pone.0135074)
Supplement: S4 Fig — The top left panel shows tumor CNA data, and the color key indicates the number of copies. The bottom left panel shows the heatmap of the heatmap of LOH data (magenta, LOH present; blue, no LOH; white, not informative). In both heatmaps, the rows stand for SNPs and the columns stand for samples. Based on the CNV data, we cluster the samples into two groups (13 patients in the red group and 62 patients in the blue group) using hierarchical clustering algorithm. The right panel shows the Cumulative Incidence curves of the OSCC-specific death of the patients in the two clusters. The X-axis indicates the years between surgery and last follow-up or death. And the Y-axis indicates the mortality rate. (DOCX) [file pone.0135074.s004.docx]

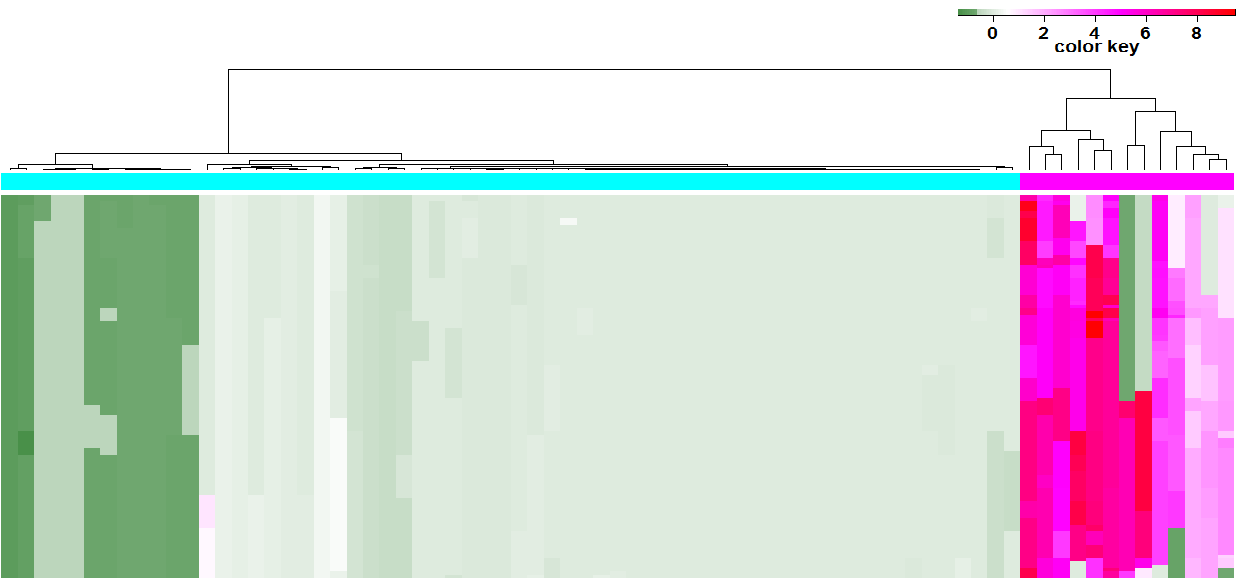

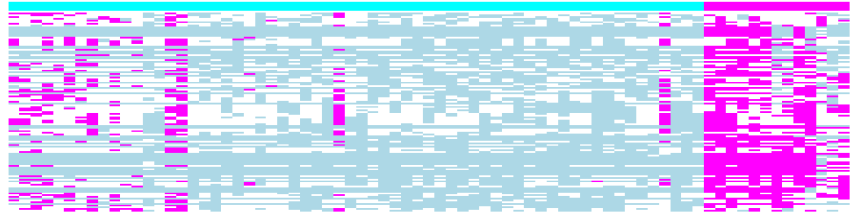

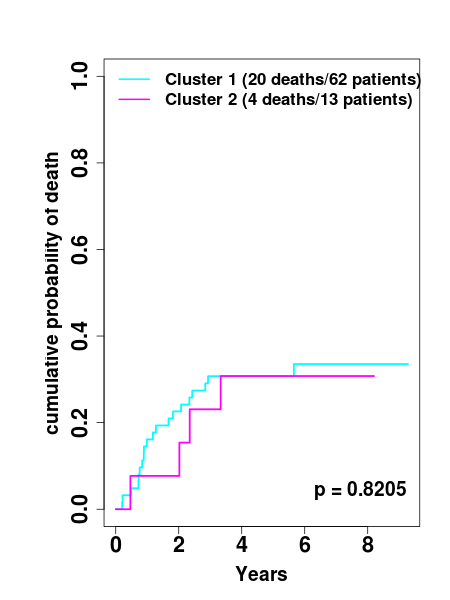


**Figure S4:** CNA and LOH data for the high-level amplification region 2 on Chr. 11 (115 probes from 11q22-24 with nucleotide position from nt99440128 to nt102804260). The **top left panel** shows tumor CNA data, and the color key indicates the number of copies. The **bottom left panel** shows the heatmap of the heatmap of LOH data (magenta, LOH present; blue, no LOH; white, not informative). In both heatmaps, the rows stand for SNPs and the columns stand for samples. Based on the CNV data, we cluster the samples into two groups (13 patients in the red group and 62 patients in the blue group) using hierarchical clustering algorithm. **The right panel** shows the Cumulative Incidence curves of the OSCC-specific death of the patients in the two clusters. The X-axis indicates the years between surgery and last follow-up or death. And the Y-axis indicates the mortality rate.
