# Supplementary material for: Genome-Wide Loss of Heterozygosity and DNA Copy Number Aberration in HPV-Negative Oral Squamous Cell Carcinoma and Their Associations with Disease-Specific Survival
Source: PLoS One. 2015 Aug 6;10(8):e0135074. doi: 10.1371/journal.pone.0135074 (PMC4527746; doi:10.1371/journal.pone.0135074)
Supplement: S5 Fig — The top left panel shows the heatmap of LOH data (magenta, LOH present; blue, no LOH; white, not informative). The bottom left panel shows the heatmap of tumor CNA data, and the color key indicates the copy number. In both heatmaps, the rows stand for SNPs and the columns stand for samples. Based on the LOH data, we clustered the samples into two groups (24 patients in the magenta group and 51 patients in the blue group) using hierarchical clustering algorithm. The right panel shows the Cumulative Incidence curves of the OSCC-specific death of the patients in the two clusters. The X-axis indicates the years between surgery and last follow-up or death. The Y-axis indicates the mortality rate. (DOCX) [file pone.0135074.s005.docx]

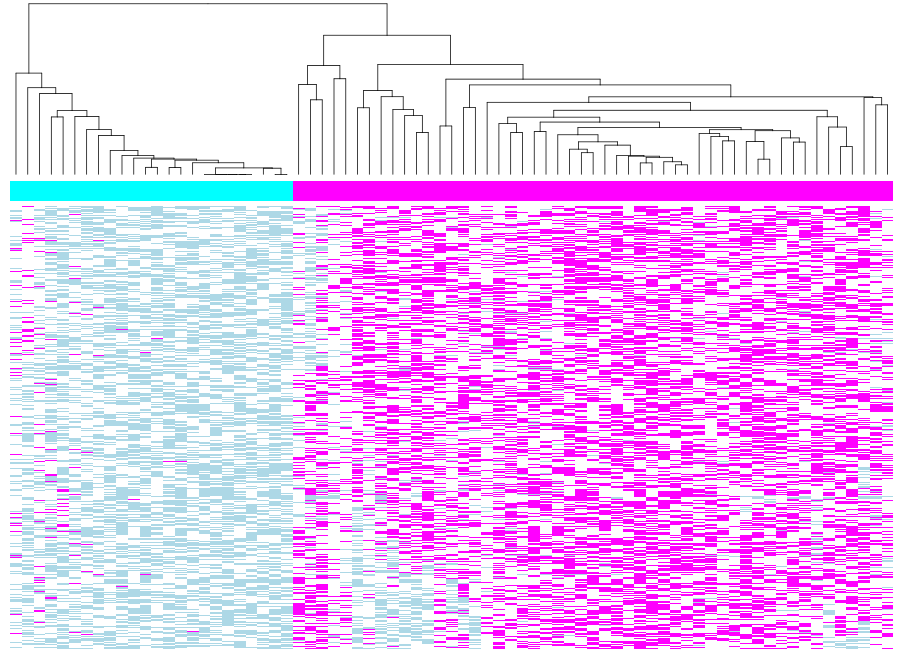

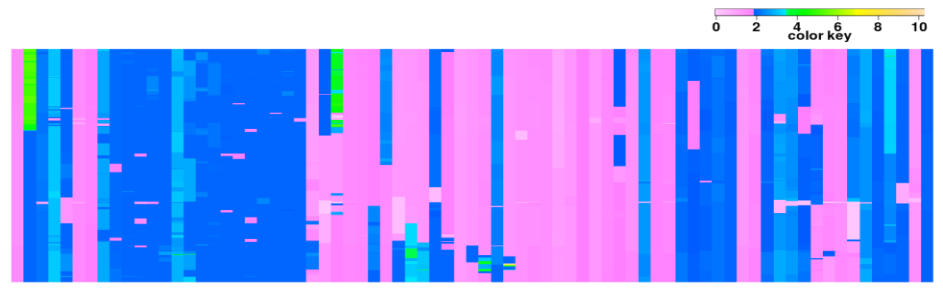

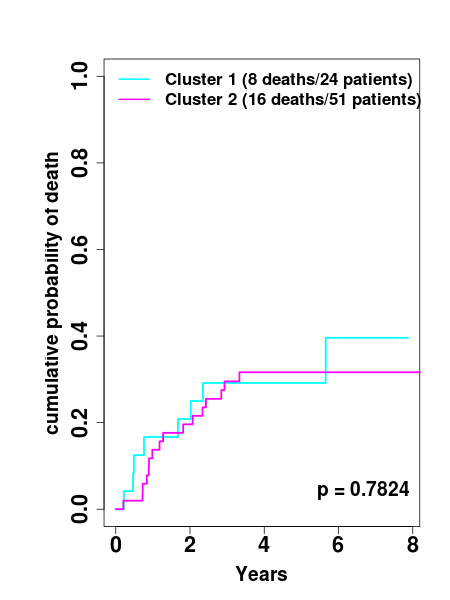


**Figure S5:** LOH and CNA data for Chr. 9p with frequent LOH event (2579 probes from nucleotide position from 36587 to 38761831). The **top left panel** shows the heatmap of LOH data (magenta, LOH present; blue, no LOH; white, not informative). The **bottom left panel** shows the heatmap of tumor CNA data, and the color key indicates the copy number. In both heatmaps, the rows stand for SNPs and the columns stand for samples. Based on the LOH data, we clustered the samples into two groups (24 patients in the magenta group and 51 patients in the blue group) using hierarchical clustering algorithm. **The right panel** shows the Cumulative Incidence curves of the OSCC-specific death of the patients in the two clusters. The X-axis indicates the years between surgery and last follow-up or death. The Y-axis indicates the mortality rate.
