# Supplementary material for: Genome-Wide Loss of Heterozygosity and DNA Copy Number Aberration in HPV-Negative Oral Squamous Cell Carcinoma and Their Associations with Disease-Specific Survival
Source: PLoS One. 2015 Aug 6;10(8):e0135074. doi: 10.1371/journal.pone.0135074 (PMC4527746; doi:10.1371/journal.pone.0135074)
Supplement: S6 Fig — The top left panel shows the heatmap of tumor CN data, and the color key indicates the copy number. The bottom left panel shows the heatmap of LOH data (magenta, LOH present; blue, no LOH; white, not informative). In both heatmaps, the rows stand for SNPs and the columns stand for samples. Based on the CNA data, we clustered the samples into two groups (15 patients in the magenta group and 60 patients in the blue group) using hierarchical clustering algorithm. The right panel shows the Cumulative Incidence curves of the OSCC-specific death of the patients in the two clusters. The X-axis indicates the years between surgery and last follow-up or death. The Y-axis indicates the mortality rate. Survival of the two clusters of patients was significantly different (p = 0.199) according to log rank test. (DOCX) [file pone.0135074.s006.docx]

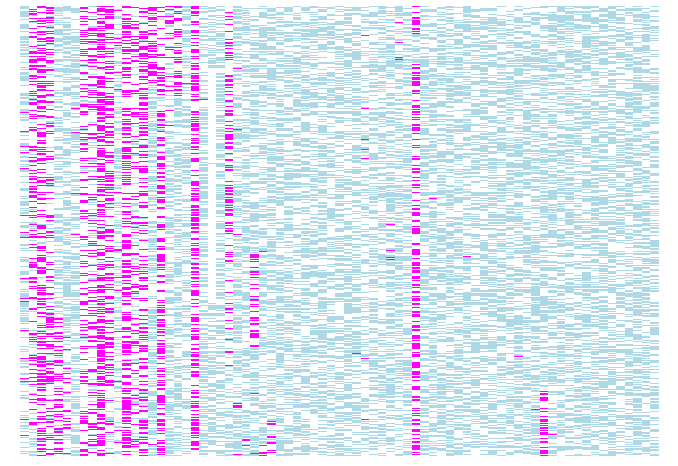

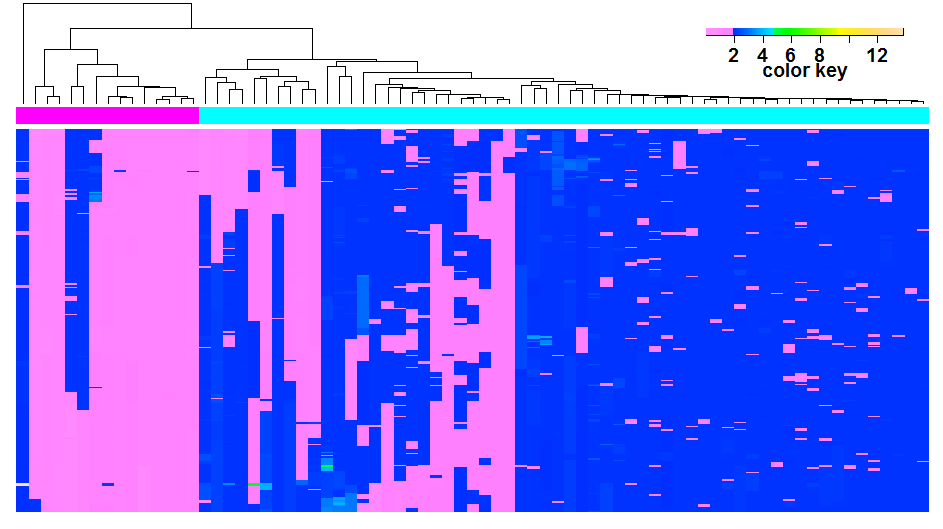

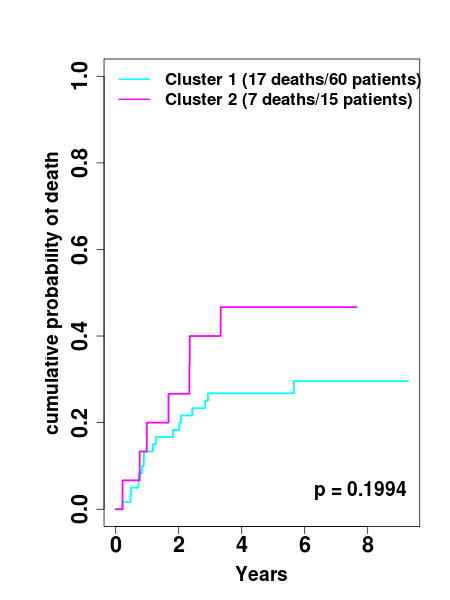


**Figure S6:** CNA and LOH data for the whole Chr. 4q region (8975 probes from nucleotide position from 63637813 to 183550869). The **top left panel** shows the heatmap of tumor CN data, and the color key indicates the copy number. The **bottom left panel** shows the heatmap of LOH data (magenta, LOH present; blue, no LOH; white, not informative). In both heatmaps, the rows stand for SNPs and the columns stand for samples. Based on the CNA data, we clustered the samples into two groups (15 patients in the magenta group and 60 patients in the blue group) using hierarchical clustering algorithm. **The right panel** shows the Cumulative Incidence curves of the OSCC-specific death of the patients in the two clusters. The X-axis indicates the years between surgery and last follow-up or death. The Y-axis indicates the mortality rate. Survival of the two clusters of patients was significantly different (p=0.199) according to log rank test.
