# Supplementary material for: Genome-Wide Loss of Heterozygosity and DNA Copy Number Aberration in HPV-Negative Oral Squamous Cell Carcinoma and Their Associations with Disease-Specific Survival
Source: PLoS One. 2015 Aug 6;10(8):e0135074. doi: 10.1371/journal.pone.0135074 (PMC4527746; doi:10.1371/journal.pone.0135074)
Supplement: S1 Table — (DOCX) [file pone.0135074.s007.docx]

**Table S1.** Selected characteristics of HPV-negative oral squamous cell cancer patients, University of Washington Affiliated Institutions, 2004-2010 (n=75)

| **Characteristic** | **n** | **(%)** |
| --- | --- | --- |
| **Age at diagnosis (years)** |  |  |
| 23-50 | 18 | (24.0) |
| 50-59 | 23 | (30.7) |
| 60-69 | 19 | (25.3) |
| 70-85 | 15 | (20.0) |
| **Sex** |  |  |
| M | 47 | (62.7) |
| F | 28 | (37.3) |
| **Race** |  |  |
| White | 72 | (96.0) |
| Non-white | 3 | (4.0) |
| **Smoking history** |  |  |
| Never | 16 | (21.3) |
| Former | 26 | (34.7) |
| Current | 33 | (44.0) |
| **Alcohol use history** |  |  |
| Never | 2 | (2.7) |
| Former | 21 | (28.8) |
| Current | 50 | (68.5) |
| Unknown | 2 |  |
| **Tumor site** |  |  |
| Oral cavity | 70 | (93.3) |
| Oropharynx | 5 | (6.7) |
| **T Stage** |  |  |
| T1/T2 | 45 | (60.8) |
| T3/T4 | 29 | (39.2) |
| Unknown | 1 |  |
| **N Stage** |  |  |
| N0 | 40 | (53.3) |
| N1 | 35 | (46.7) |
| **AJCC Stage** |  |  |
| I | 20 | (27.2) |
| II | 10 | (13.5) |
| III | 8 | (10.8) |
| IV | 36 | (48.6) |
| Unknown | 1 |  |
