# Supplementary material for: Genome-Wide Loss of Heterozygosity and DNA Copy Number Aberration in HPV-Negative Oral Squamous Cell Carcinoma and Their Associations with Disease-Specific Survival
Source: PLoS One. 2015 Aug 6;10(8):e0135074. doi: 10.1371/journal.pone.0135074 (PMC4527746; doi:10.1371/journal.pone.0135074)
Supplement: S3 Table — (DOCX) [file pone.0135074.s009.docx]

**Table S3.** Selected characteristics of OSCC patients according to clusters defined by genome-wide tumor CNA or LOH events, University of Washington Affiliated Institutions, 2004-2010

|  | **By genome-wide CNA** | | | | | **By genome-wide LOH** | | | | | |
| --- | --- | --- | --- | --- | --- | --- | --- | --- | --- | --- | --- |
|  | **Cluster 1**  **n (%)** | | **Cluster 2**  **n (%)** | | **p-value** | **Cluster 1**  **n (%)** | | | **Cluster 2**  **n (%)** | | **p-value** |
| **Tumor Site** |  |  |  |  |  |  |  |  | |  |  |
| Oral cavity | 58 | (93.5) | 12 | (92.3) | 1.0 | 41 | (95.3) | 29 | | (90.6) | 0.645 |
| Oropharynx | 4 | (6.5) | 1 | (7.7) |  | 2 | (4.7) | 3 | | (9.4) |  |
| **T stage** |  |  |  |  |  |  |  |  | |  |  |
| T1/T2 | 39 | (62.9) | 6 | (50.0) | 0.118 | 26 | (61.9) | 19 | | (59.4) | 1.0 |
| T3/T4 | 23 | (37.1) | 6 | (50.0) |  | 16 | (38.1) | 13 | | (40.6) |  |
| Unknown | 0 |  | 1 |  |  | 1 |  | 0 | |  |  |
| **N stage** |  |  |  |  |  |  |  |  | |  |  |
| N0 | 31 | (50.0) | 9 | (69.2) | 0.238 | 26 | (60.5) | 14 | | (43.8) | 0.168 |
| N1 | 31 | (50.0) | 4 | (30.8) |  | 17 | (39.5) | 18 | | (56.3) |  |
| **AJCC stage** |  |  |  |  |  |  |  |  | |  |  |
| I | 16 | (25.8) | 4 | (33.3) | 0.410 | 11 | (26.2) | 9 | | (28.1) | 0.866 |
| II | 9 | (14.5) | 1 | (8.3) |  | 7 | (16.7) | 3 | | (9.4) |  |
| III | 7 | (11.3) | 1 | (8.3) |  | 5 | (11.9) | 3 | | (9.4) |  |
| IV | 30 | (48.4) | 6 | (50.0) |  | 19 | (45.2) | 17 | | (53.1) |  |
| Unknown | 0 |  | 1 |  |  | 1 |  | 0 | |  |  |
| **Smoking history** |  |  |  |  |  |  |  |  | |  |  |
| Current | 25 | (40.3) | 8 | (61.5) | 0.091 | 16 | (37.2) | 17 | | (53.1) | 0.007 |
| Former | 21 | (33.9) | 5 | (38.5) |  | 21 | (48.8) | 5 | | (15.6) |  |
| Never | 16 | (25.8) | 0 | (0.0) |  | 6 | (14.0) | 10 | | (31.3) |  |
| **Alcohol use history** |  |  |  |  |  |  |  |  | |  |  |
| Current | 44 | (72.1) | 6 | (50.0) | 0.151 | 28 | (66.7) | 22 | | (71.0) | 0.949 |
| Former | 15 | (24.6) | 6 | (50.0) |  | 13 | (31.0) | 8 | | (25.8) |  |
| Never | 2 | (3.3) | 0 | (0.0) |  | 1 | (2.4) | 1 | | (3.2) |  |
| Unknown | 1 |  | 1 |  |  | 1 |  | 1 | |  |  |
