# Supplementary material for: Genome-Wide Loss of Heterozygosity and DNA Copy Number Aberration in HPV-Negative Oral Squamous Cell Carcinoma and Their Associations with Disease-Specific Survival
Source: PLoS One. 2015 Aug 6;10(8):e0135074. doi: 10.1371/journal.pone.0135074 (PMC4527746; doi:10.1371/journal.pone.0135074)
Supplement: S4 Table — (DOCX) [file pone.0135074.s010.docx]

**Table S4.** Selected characteristics for patients in clusters defined by CNA in Chr. 11, Region 1

(11q13.1-14.3, nt 68683098-nt 70375682) and Region 2 (11q22-24, nt99440128-nt102804260)

|  | **Region 1** | | | | | **Region 2** | | | | | |
| --- | --- | --- | --- | --- | --- | --- | --- | --- | --- | --- | --- |
|  | **Cluster 1**  **n (%)** | | **Cluster 2**  **n (%)** | | **p-value** | **Cluster 1**  **n (%)** | | | **Cluster 2**  **n (%)** | | **p-value** |
| **Tumor Site** |  |  |  |  |  |  |  |  | |  |  |
| Oral cavity | 50 | (96.2) | 20 | (87.0) | 0.165 | 59 | (95.2) | 11 | | (84.6) | 0.205 |
| Oropharynx | 2 | (3.8) | 3 | (13.0) |  | 3 | (4.8) | 2 | | (15.4) |  |
| **T stage** |  |  |  |  |  |  |  |  | |  |  |
| T1/T2 | 30 | (58.8) | 15 | (65.2) | 0.860 | 37 | (60.7) | 8 | | (61.5) | 1.0 |
| T3/T4 | 21 | (41.2) | 8 | (34.8) |  | 24 | (39.3) | 5 | | (38.5) |  |
| Unknown | 1 |  | 0 |  |  | 1 |  | 0 | |  |  |
| **N stage** |  |  |  |  |  |  |  |  | |  |  |
| N0 | 28 | (53.8) | 12 | (52.2) | 1.0 | 33 | (53.2) | 7 | | (53.8) | 1.0 |
| N1 | 24 | (46.2) | 11 | (47.8) |  | 29 | (46.8) | 6 | | (46.2) |  |
| **AJCC stage** |  |  |  |  |  |  |  |  | |  |  |
| I | 12 | (23.5) | 8 | (34.8) | 0.448 | 15 | (24.6) | 5 | | (38.5) | 0.835 |
| II | 8 | (15.7) | 2 | (8.7) |  | 9 | (14.8) | 1 | | (7.7) |  |
| III | 4 | (7.8) | 4 | (17.4) |  | 7 | (11.5) | 1 | | (7.7) |  |
| IV | 27 | (52.9) | 9 | (39.1) |  | 30 | (49.2) | 6 | | (46.2) |  |
| Unknown | 1 |  | 0 |  |  | 1 |  | 0 | |  |  |
| **Smoking history** |  |  |  |  |  |  |  |  | |  |  |
| Current | 19 | (36.5) | 14 | (60.9) | 0.157 | 30 | (48.4) | 3 | | (23.1) | 0.067 |
| Former | 20 | (38.5) | 6 | (26.1) |  | 22 | (35.5) | 4 | | (30.8) |  |
| Never | 13 | (25.0) | 3 | (13.0) |  | 10 | (16.1) | 6 | | (46.2) |  |
| **Alcohol use history** |  |  |  |  |  |  |  |  | |  |  |
| Current | 32 | (62.7) | 18 | (81.8) | 0.168 | 42 | (70.0) | 8 | | (61.5) | 0.774 |
| Former | 18 | (35.3) | 3 | (13.6) |  | 16 | (26.7) | 5 | | (38.5) |  |
| Never | 1 | (2.0) | 1 | (4.5) |  | 2 | (3.3) | 0 | | (0.0) |  |
| Unknown | 1 |  | 1 |  |  | 2 |  | 0 | |  |  |
