# Supplementary material for: Genome-Wide Loss of Heterozygosity and DNA Copy Number Aberration in HPV-Negative Oral Squamous Cell Carcinoma and Their Associations with Disease-Specific Survival
Source: PLoS One. 2015 Aug 6;10(8):e0135074. doi: 10.1371/journal.pone.0135074 (PMC4527746; doi:10.1371/journal.pone.0135074)
Supplement: S5 Table — (DOCX) [file pone.0135074.s011.docx]

**Table S5.** Selected characteristics of patients in clusters defined by LOH on Chr. 9p

|  | **Cluster 1**  **n (%)** | | **Cluster 2**  **n (%)** | | **p-value** |
| --- | --- | --- | --- | --- | --- |
| **Tumor Site** |  |  |  |  |  |
| Oral cavity | 24 | (100.0) | 46 | (90.2) | 0.170 |
| Oropharynx | 0 | (0.0) | 5 | (9.8) |  |
| **T stage** |  |  |  |  |  |
| T1/T2 | 14 | (58.3) | 31 | (62.0) | 0.866 |
| T3/T4 | 10 | (41.7) | 19 | (38.0) |  |
| Unknown | 0 |  | 1 |  |  |
| **N stage** |  |  |  |  |  |
| N0 | 12 | (50.0) | 28 | (54.9) | 0.805 |
| N1 | 12 | (50.0) | 23 | (45.1) |  |
| **AJCC stage** |  |  |  |  |  |
| I | 9 | (37.5) | 11 | (22.0) | 0.203 |
| II | 1 | (4.2) | 9 | (18.0) |  |
| III | 1 | (4.2) | 7 | (14.0) |  |
| IV | 13 | (54.2) | 23 | (46.0) |  |
| Unknown | 0 |  | 1 |  |  |
| **Smoking history** |  |  |  |  |  |
| Current | 4 | (16.7) | 29 | (56.9) | 0.002 |
| Former | 11 | (45.8) | 15 | (29.4) |  |
| Never | 9 | (37.5) | 7 | (13.7) |  |
| **Alcohol use history** |  |  |  |  |  |
| Current | 15 | (62.5) | 35 | (71.4) | 0.625 |
| Former | 8 | (33.3) | 13 | (26.5) |  |
| Never | 1 | (4.2) | 1 | (2.0) |  |
| Unknown | 0 |  | 2 |  |  |
