# Supplementary material for: Genome-Wide Loss of Heterozygosity and DNA Copy Number Aberration in HPV-Negative Oral Squamous Cell Carcinoma and Their Associations with Disease-Specific Survival
Source: PLoS One. 2015 Aug 6;10(8):e0135074. doi: 10.1371/journal.pone.0135074 (PMC4527746; doi:10.1371/journal.pone.0135074)
Supplement: S6 Table — (DOCX) [file pone.0135074.s012.docx]

**Table S6.** Selected characteristics for patients in clusters defined by LOH on Chr. 4q

|  | **Cluster 1**  **n (%)** | | **Cluster 2**  **n (%)** | | **p-value** |
| --- | --- | --- | --- | --- | --- |
| **Tumor Site** |  |  |  |  |  |
| Oral cavity | 58 | (95.1) | 12 | (85.7) | 0.232 |
| Oropharynx | 3 | (4.9) | 2 | (14.3) |  |
| **T stage** |  |  |  |  |  |
| T1/T2 | 37 | (60.7) | 8 | (61.5) | 0.240 |
| T3/T4 | 24 | (39.3) | 5 | (38.5) |  |
| Unknown | 0 |  | 1 |  |  |
| **N stage** |  |  |  |  |  |
| N0 | 33 | (54.1) | 7 | (50.0) | 1.0 |
| N1 | 28 | (45.9) | 7 | (50.0) |  |
| **AJCC stage** |  |  |  |  |  |
| I | 14 | (23.0) | 6 | (46.2) | 0.048 |
| II | 10 | (16.4) | 0 | (0.0) |  |
| III | 8 | (13.1) | 0 | (0.0) |  |
| IV | 29 | (47.5) | 7 | (53.8) |  |
| Unknown | 0 |  | 1 |  |  |
| **Smoking history** |  |  |  |  |  |
| Current | 27 | (44.3) | 6 | (42.9) | 1.0 |
| Former | 21 | (34.4) | 5 | (35.7) |  |
| Never | 13 | (21.3) | 3 | (21.4) |  |
| **Alcohol use history** |  |  |  |  |  |
| Current | 43 | (72.9) | 7 | (50.0) | 0.228 |
| Former | 14 | (23.7) | 7 | (50.0) |  |
| Never | 2 | (3.4) | 0 | (0.0) |  |
| Unknown | 2 |  | 0 |  |  |
